# Numerical Investigation of the Water-Exit Performance of a Bionic Unmanned Aerial-Underwater Vehicle with Front-Mounted Propeller

**DOI:** 10.3390/biomimetics11010021

**Published:** 2025-12-31

**Authors:** Yu Dong, Qigan Wang, Wei Wu, Zhijun Zhang

**Affiliations:** 1 Key Laboratory of CNC Equipment Reliability (Ministry of Education) and School of Mechanical and Aerospace Engineering, Jilin University, Changchun 130022, China; 2 School of Mechanical Engineering, University of Shanghai for Science & Technology, Shanghai 200093, China

**Keywords:** water exit, unmanned aerial-underwater vehicle, bionic model, multiphase flow

## Abstract

This work presents a numerical study of the water-exit characteristics of a bioinspired unmanned aerial-underwater vehicle (UAUV) equipped with a front-mounted propeller. A robust solution framework was established on the basis of a modified Shear Stress Transport (SST) turbulence model, volume of fluid (VOF) multiphase formulation, overset grid technique, and six degrees of freedom (6-DOF) motion model; the framework was verified against a canonical water-exit case of a sphere. Inspired by the morphology and water-exit behavior of flying fish, a bioinspired three-dimensional (3D) model was designed. Using this framework, the effects of the front-mounted propeller configuration, exit velocity, and exit angle were examined; the exit process under different conditions was analyzed; and the relationship between exit drag and exit state was quantified. The results demonstrate that the proposed approach can resolve the water-exit performance of the bioinspired UAUV in detail. Folding the front-mounted propeller effectively reduces exit drag and mitigates high-pressure concentrations on the blades. When the exit velocity is ≥8 m/s and the exit angle θ ≤ 30°, the peak exit drag does not surpass 90.004 N. The peak exit drag exhibits a pronounced quadratic relationship with both exit velocity and exit angle. To ensure safe water exit, the UAUV should avoid exiting with the front-mounted propeller deployed and avoid excessively low exit velocities and overly large exit angles. The numerical investigation of exit drag provides effective bioinspired design guidelines and a feasible analysis strategy for UAUV development. In conclusion, the findings provide crucial insights for designing more efficient bioinspired UAUVs, particularly in terms of minimizing water-exit drag and optimizing the configuration of the front-mounted propeller.

## 1. Introduction

The oceans are the largest bodies of water on Earth, covering approximately 360 million km^2^—about 71% of the planet’s surface. Nevertheless, our understanding of the oceans remains limited. The unmanned aerial-underwater vehicle (UAUV) is a platform capable of seamlessly transitioning between flight and underwater operation. It can switch from an unmanned aerial vehicle to an unmanned underwater vehicle, delivering favorable aerodynamic or hydrodynamic performance in the corresponding medium while maintaining low observability [1]. Such systems can carry out missions in complex and rapidly changing environments—such as maritime search and rescue, environmental monitoring, and polar scientific expeditions—that are often difficult or hazardous for human operators. This technology shows broad application potential in both defense and civilian domains.

Over the long course of natural selection, animals have evolved distinctive morphologies and functions that provide valuable inspiration for technological innovation [2]. In recent years, propelled by rapid advances in engineering, studies on bioinspired and morphing UAUVs have increased markedly. Bioinspired UAUVs derive their design cues from animals capable of operating across different media—such as plunge-diving seabirds and flying fish—by emulating their water-entry or water-exit behaviors. Morphing UAUVs, by contrast, actively reconfigure their geometry during entry or exit—adjusting structures such as wings or propellers—to optimize performance.

Bioinspired UAUVs typically feature high-aspect-ratio planforms, nearly elliptical lift distributions, and elevated underwater lift-to-drag ratios, whereas morphing UAUVs mitigate structural damage by reconfiguring elements to avoid water-entry impact loads or excessive water-exit drag. Consequently, many researchers have sought to hybridize these two UAUV paradigms to achieve superior overall performance. The bioinspired UAUV with a front-mounted propeller investigated in this study is one such cross-medium unmanned vehicle that integrates attributes of both bioinspired and morphing designs.

The drag generated during a UAUV’s water exit is tightly coupled to interactions among the vehicle, water, and air. Because the physical properties of water and air differ substantially—the density and dynamic viscosity of water are approximately 800 and 60 times those of air, respectively [3]—these contrasts exert a significant influence on water-exit performance. To accurately characterize the drag evolution during the water exit of a bioinspired UAUV with a front-mounted propeller and to prevent drag-induced structural damage, it is therefore necessary to identify and analyze the factors governing water-exit drag for such vehicles.

Research on the water-exit motion of bodies with different shapes has largely relied on experimental observations and multiphase-flow simulations. Experimentally, Siddall et al. [4] designed a novel amphibious aircraft by simplifying the morphology of gannets and analyzed how exit attitude affects water-exit performance. Wu et al. [5] investigated the water-exit and re-entry of rotating bodies through combined experiments and simulations, examining the effects of initial submergence ratio and density on hydrodynamics. Shi et al. [6] experimentally studied cavitation mechanisms and motion characteristics of underwater-launched projectiles, quantifying how launch speed and lateral velocity influence cavity evolution and dynamics. Zhang et al. [7] explored how high-pressure exhaust conditions affect the dynamic evolution of cavities during fully free water exit. Han et al. [8] conducted experiments on the water-exit process of a foldable-wing vehicle under varying speeds and angles. Qu et al. [9] experimentally characterized the dynamic evolution of cavities formed at the muzzle of the launch tube during projectile water exit.

On the numerical side, Chu et al. [10] simulated a cylinder’s water-exit process, capturing free-surface interactions and analyzing velocity and acceleration histories. Cao et al. [11] simulated the underwater trajectory of a vertically launched submersible and computed the associated flow field during water exit, assessing how pressure variations influence the exit trajectory. Tassin et al. [12] employed analytical and numerical models to study water entry and water exit of bodies whose properties vary in time within a two-dimensional medium. Wang et al. [13] developed a three-dimensional trajectory model for submersibles based on dynamic-mesh technology, simulating the water exit of submarine-launched missiles under specified conditions and analyzing their underwater ballistics and attitude angles. Moshari et al. [14] built a dynamic-mesh model for a cylinder’s water exit using a volume of fluid formulation within a finite-volume framework. Hu et al. [15] established a hydrodynamic model for an oblique water exit of a morphing unmanned aircraft vehicle and analyzed its exit process. Ni and Wu [16] combined the boundary element method (BEM) with slender body theory (SBT) to investigate the vertical water-exit of a buoyant sphere. Ma et al. [17] simulated the water entry and water exit of a slender vehicle, obtaining its displacement and pitch evolution and revealing the effect of angular velocity on the underwater trajectory. Liu et al. [18] numerically examined the water exit of a diving vehicle under different initial pitch angles and speeds, quantifying their effects on exit motion. Guo et al. [19] used VOF interface capturing with overset grids to compute gas–liquid interactions during high-speed, oblique cylinder water exit, analyzing cavitation evolution, pressure distribution, and the influence of exit angle on surface pressure and drag. Zhou et al. [20] numerically studied the temporal variations in kinematic and dynamic parameters of a manta ray’s water exit and elucidated the evolution of vortex structures during its underwater phase. Sun et al. [21] addressed attitude-stability issues of a vectored dual-rotor vehicle during water exit using multiphase-flow simulations, deriving laws for free-surface effects on flow-field changes at different exit heights. Huang et al. [22] employed multiphase simulations coupled with large eddy simulation (LES) of turbulence to investigate the dynamics and energetics of a neutrally buoyant sphere’s water exit. He et al. [23] conducted aerodynamic and hydrodynamic studies on the water-crossing problem of a bioinspired amphibious unmanned aerial vehicle and proposed a conceptual design featuring staged, multi-mode motion for traversing aquatic environments. To address the low efficiency of the classical oblique locomotion trajectory, Gan et al. [24] proposed an improved oblique trajectory scheme based on the joint optimization of stroke angle and angle-of-attack profiles, which was validated through simulation and experiments.

To date, water-exit modes have been extensively studied. However, for UAUVs, devising effective structural designs and developing accurate predictive models for water-exit drag remains challenging. Consequently, in-depth investigation of biological morphology and its linkage to water-exit performance is of substantial importance for understanding water-exit processes and advancing UAUV design.

This study investigates the behavior of a bionic unmanned aerial-underwater vehicle equipped with a front-mounted propeller (hereinafter referred to as the bionic UAUV) as it traverses the air-water interface. The innovation lies in combining a foldable front propeller with a bioinspired design. Numerical simulations of the bionic UAUV’s water-exit process were carried out to evaluate its performance. A detailed analysis of the process elucidated the general relationships between water-exit performance and exit velocity/angle, providing valuable references for future structural design and predictive modeling of exit drag. First, an unsteady numerical approach based on the SST *k-ω* turbulence model and a VOF interface-capturing scheme is validated against a canonical sphere water-exit scenario. Next, salient morphological features of flying fish are extracted to design a UAUV architecture suitable for airborne flight, underwater navigation, and water exit. Subsequently, unsteady multiphase simulations are performed to study the bionic UAUV’s water-exit process and analyze its performance under representative flow conditions. Finally, the effects of exit velocity and exit angle on the water-exit dynamics are assessed. The structure of this paper is as follows: Section 2 details the methods, including the numerical model and validation approach; Section 3 presents the results and discusses the impact of various design factors on the water-exit performance; and Section 4 provides a conclusion and outlines the implications for future UAUV design and optimization.

## 2. Methods

### 2.1. SST Turbulence Model

The SST *k-ω* turbulence model exhibits excellent adaptability, particularly for rotation-dominated flows and shear-layer dynamics. Its blending formulation effectively reduces the free-stream sensitivity of the standard *k-ω* model while preserving high near-wall resolution. A principal advantage of the model is the incorporation of the vorticity magnitude into the turbulence production term, enabling an anisotropic response to rotational strain.

The SST *k-ω* turbulence model adopts the blending functions proposed by Menter together with a modified eddy-viscosity formulation [25]. Its transport equations incorporate a vorticity-based production term and the turbulence viscosity, where the vorticity magnitude is defined as $\Omega \left(\Omega =\sqrt{2}\Omega_{ij}\right)$. A concise overview is as follows:

(1) $$\frac{\partial \left(\rho k\right)}{\partial t}+\frac{\partial \left(\rho u_{j}k\right)}{\partial x_{j}}=P_{k}-\beta^{\prime }\rho k\omega +\frac{\partial }{\partial x_{j}}\left[\left(\mu +\frac{\mu_{T}}{\sigma_{k}}\right)\frac{\partial k}{\partial x_{j}}\right]$$

(2) $$\frac{\partial \left(\rho \omega \right)}{\partial t}+\frac{\partial \left(\rho u_{j}\omega \right)}{\partial x_{j}}=P_{\omega }-\beta \rho \omega^{2}+\frac{\partial }{\partial x_{j}}\left[\left(\mu +\frac{\mu_{T}}{\sigma_{k}}\right)\frac{\partial \omega }{\partial x_{j}}\right]+2\left(1-2F_{1}\right)\frac{\rho }{\sigma_{\omega_{2}}\omega }\frac{\partial k}{\partial x_{j}}\frac{\partial \omega }{\partial x_{j}}$$

Here, ρ is the fluid density. k is the turbulent kinetic energy. $u_{j}$ represents the velocity components in the Cartesian coordinate system (j = 1, 2, or 3). $P_{k}$ denotes the source term of k. $\beta^{\prime }$ are the second model constants in the SST formulation. ω is the specific dissipation rate. μ is the molecular (laminar) viscosity. $\mu_{T}$ is the turbulent viscosity. $P_{\omega }$ denotes the source term of ω. β is the first model constant in the SST formulation. $\sigma_{k}$ is the turbulent kinetic energy constant. $F_{1}$ is the first blending function. $\sigma_{\omega }$ and $\sigma_{\omega_{2}}$ are the first and second specific dissipation rate constants.

Based on the concepts of Reynolds stress and flow separation, modifications were made to a structured-grid RANS solver that uses Menter’s SST *k-ω* turbulence model. These modifications improve the accuracy of flow-separation predictions and enhance the adaptability of the Reynolds-stress formulation.

The turbulent viscosity and the turbulent kinetic energy are computed as follows:

(3) $$\mu_{T}=min\left[\frac{\rho k}{\omega^{\prime }}\frac{a_{1}\rho k}{\Omega F_{2}}\right]$$

(4) $$P_{k}=\mu_{T}\Omega^{2}-\frac{2}{3}\rho k\delta_{ij}\frac{\partial u_{i}}{\partial x_{j}}$$

$F_{2}$ represents the second blending function, and Ω represents the magnitude of the vorticity.

Using a separation-prediction strategy (i.e., a separation-based approach), a correction was introduced to the specific dissipation rate. Under low-speed airborne flight and underwater navigation conditions, an incompressible-flow assumption was adopted to simplify density-dependent terms. Further details of the model can be found in Gan [26] and Gan & Zhang [27].

### 2.2. Volume of Fluid (VOF) Model

The volume of fluid (VOF) model is an interface-capturing approach used to represent the interface between two or more immiscible fluids [28,29]. The method introduces a volume fraction function C that satisfies a transport equation; C denotes the ratio of the tracked fluid’s volume to the volume of a computational cell. Cells with C = 0 contain none of the tracked fluid, whereas cells with C = 1 are completely filled by it; interfacial cells satisfy 0 < C < 1. By solving the transport equation for C at each time instant, the spatial distribution of the volume fraction over the computational domain is obtained, thereby enabling interface capture.

Assume the computational domain Ω is composed of the subdomains $\Omega^{1}$ and $\Omega^{2}$ occupied by fluids A and B, respectively. Based on this domain, define the function

(5) $$\alpha \left(\vec{x},t\right)=\left\{\begin{array}{c} 1,\vec{x}\in \Omega^{1}\\ 0,\vec{x}\in \Omega^{2} \end{array}\right.$$

Within the VOF framework, the governing continuity and momentum equations for the unsteady RANS solver can be expressed as

(6) $$\frac{\partial \rho_{m}}{\partial t}+\frac{\partial }{\partial x_{i}}\left(\rho_{m}u_{i}\right)=0$$

(7) $$\frac{\partial }{\partial t}(\rho_{m}u_{i})+\frac{\partial }{\partial x_{j}}(\rho_{m}u_{i}u_{j})=-\frac{\partial p}{\partial x_{i}}+\frac{\partial }{\partial x_{i}}(-\rho_{m}{\bar{u}}_{i}{\bar{u}}_{j})+\frac{\partial }{\partial x_{j}}\left[\mu_{m}\left(\frac{\partial u_{i}}{\partial x_{j}}+\frac{\partial u_{j}}{\partial x_{i}}-\frac{2}{3}\delta_{ij}\frac{\partial u_{k}}{\partial x_{k}}\right)\right]$$

Here, the mixture density is defined as $\rho_{m}=\rho_{a}\alpha_{a}+\rho_{W}\alpha_{W}$, the dynamic viscosity is defined as $\mu_{m}=\mu_{a}\alpha_{a}+\mu_{W}\alpha_{W}$, where a and w are used as subscripts to distinguish between the air and water domains.

### 2.3. Scattering and Boundary Conditions

This study employs a numerical methodology that couples VOF interface capturing with an overset grid framework. The 3D unsteady RANS equations are discretized using a finite lower–upper symmetric Gauss–Seidel (LU-SGS) method, and pressure–velocity coupling is handled via the SIMPLEC algorithm [30,31]. The convective terms in both the momentum and turbulence transport equations are discretized with a second-order upwind scheme. The solid walls were subjected to a no-slip boundary condition, while the freestream boundaries for air and water were set as inlets and outlets, respectively. To simulate the water-exit process, a free surface boundary was defined at the air-water interface, allowing for its movement.

To ensure the convergence of the numerical simulation results, a detailed convergence study was conducted in this work. A residual criterion was set, requiring the residual values to drop below 10^−6^ in each iteration. Additionally, the simulation was considered converged when the variation in drag force was less than 1%. In all simulations performed, the changes in drag force and flow field parameters met this criterion.

### 2.4. Overset Grid

In computational fluid dynamics, an overset grid—also called a chimera or nested grid—is formed by superposing meshes of differing topology. Meshes in distinct subregions can be generated independently, with overlaps and nesting permitted between them. To enable computations across subdomains, data are exchanged at overset interfaces via interpolation. Relative motion between component grids is allowed, which makes the approach advantageous for simulations involving moving bodies. According to their role in the solution process, cells in an overset system are classified as computational (active) cells, interpolation (fringe) cells, and hole (inactive) cells.

Computational cells are used to discretize the governing equations, whereas interpolation cells acquire flow-field data from other grids during the solution process—the supplying cells are termed donor cells and the receiving cells are termed receptor cells. Hole cells do not participate in the computation; they typically lie outside the physical domain or within cut-out holes. In overset grid simulations, two key steps are hole-cutting and donor-cell search. Hole-cutting determines, for a given configuration, which cells are active and which are to be blanked.

In overset grid computations, to ensure interpolation accuracy and overall mesh quality, the characteristic cell sizes in the overlap region should be kept as consistent as possible with those of the background grid; large disparities degrade interpolation fidelity. In dynamic-mesh methods, the mesh is continually updated. By contrast, in the overset approach, the component grid within the overlap region moves with the body, eliminating the need for mesh deformation or remeshing during motion. One needs only to prescribe the body kinematics and the grid boundary conditions to simulate the hydrodynamics in the domain. This avoids numerical errors associated with mesh distortion and with cell birth/death and is therefore advantageous.

### 2.5. Six Degrees of Freedom (6-DOF) Model

The bionic UAUV possesses six degrees of freedom (6-DOF) in three-dimensional space—translations along the Cartesian x, y, and z axes and rotations about these axes. Arbitrary motions can be represented by combinations and decompositions of these DOFs. During cross-medium water entry and water exit, the vehicle is subjected to the combined effects of weight, aerodynamic forces, hydrodynamic forces, and buoyancy. The 6-DOF rigid-body translational and rotational equations of motion are given by

(8) $$m\frac{dV}{dt}=F$$

(9) $$M\frac{d\omega }{dt}=N$$

where m is the mass of the bionic UAUV; V is the translational velocity of the bionic UAUV; F is the total external force acting on the bionic UAUV, including gravity, aerodynamic force, hydrodynamic force, and buoyancy; M is the inertia tensor; ω is the angular velocity of the rigid body; and N is the total external moment (torque) acting on the bionic UAUV.

The Dynamic Fluid Body Interaction (DFBI) module in STAR-CCM+ is a powerful capability for simulating interactions between rigid bodies and fluids. It computes the forces and moments acting on a rigid body and simulates the body’s motion within the flow. Users can specify physical properties such as mass, moments of inertia, initial position, and initial velocity. The DFBI framework also accommodates multiphase problems (e.g., liquid–gas mixtures), including free-surface waves and water-impact phenomena. The 6-DOF model is implemented chiefly within the DFBI module.

### 2.6. Validation

**Figure 1:**
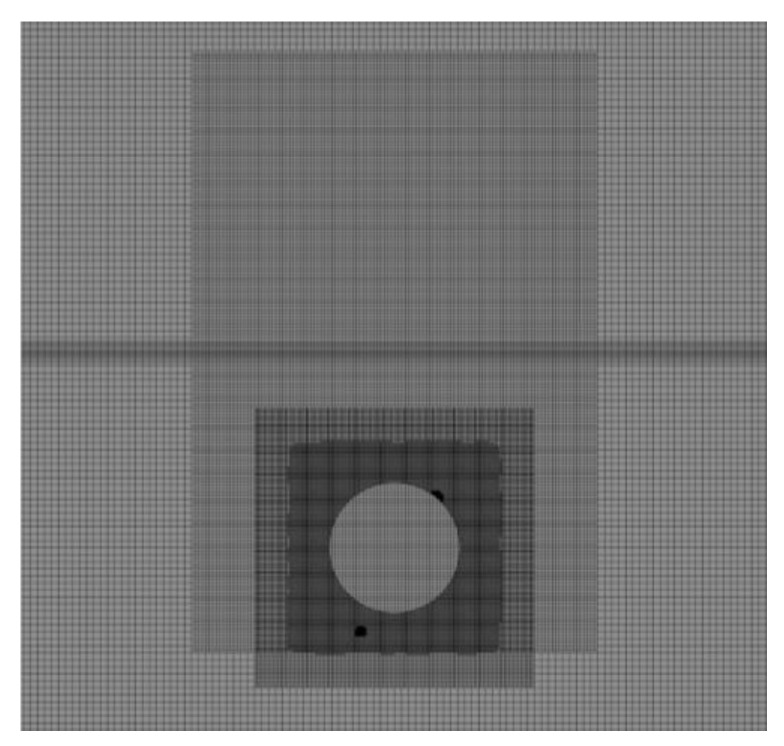
Meshing for the water-exit simulations of the sphere.

To establish the reliability and accuracy of the present simulation methodology, a numerical validation was performed for the free water exit of a sphere, and the results were compared with the experimental data of Ni et al. [32]. The model is a hollow stainless-steel sphere with a mass of 0.15 kg, a radius of 140 mm, and a density of 0.1 × 10^3^ kg m^−3^. The initial position of the sphere’s center of mass is set 210 mm below the undisturbed free surface. The mesh of the sphere is shown in Figure 1.

To more accurately simulate the solid’s water-exit process and improve numerical accuracy, mesh refinement was applied to the solid boundaries, the solid’s motion trajectory, and the air–water interface within the computational domain. Consistent with SST model guidelines, the first off-wall cell adjacent to solid surfaces was placed such that its nondimensional wall distance y^+^ lies within the valid range for standard wall functions, which is below 5 [26].

**Figure 2:**
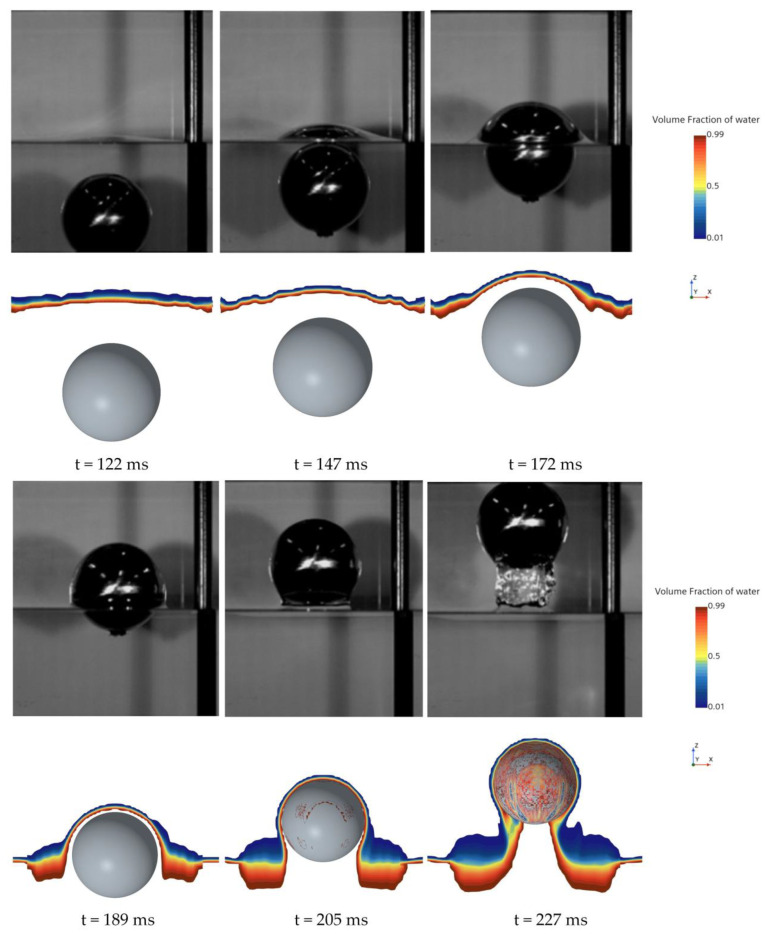
Comparison of numerical and experimental results during the sphere’s water-exit phase.

**Figure 3:**
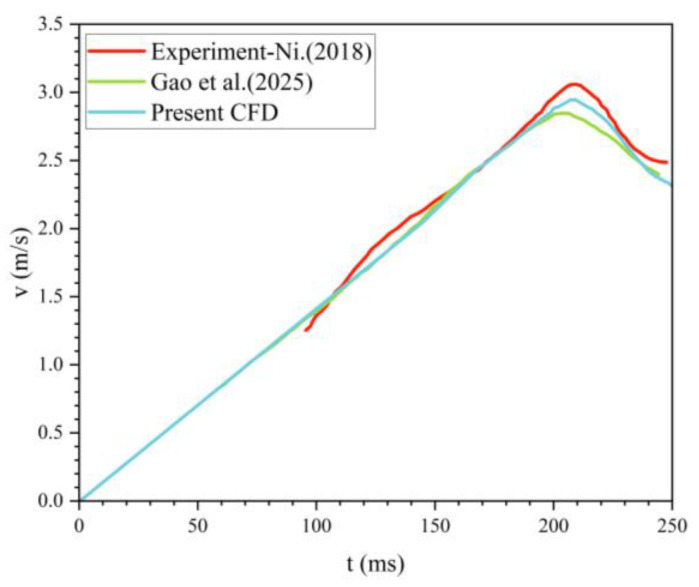
Variation of velocity during sphere water-exit over time [32,33].

Figure 2 compares six time points during the sphere’s water-exit process from experiment and simulation, showing the air–water phase distribution. In both the simulations and the experiments, the evolution of the free-surface rise during exit is clearly visible. The adopted numerical approach effectively captures fine-scale variations of the air–water interface. Figure 3 presents the sphere’s velocity–time history during water exit; the numerical results accurately reproduce the observed trend. The predicted sphere velocity agrees well with the measurements, with a maximum deviation below 5.2%, and is also consistent with the simulations of Gao et al. [33]. These results indicate that the present method can reliably predict the water-exit process of a sphere.

### 2.7. Bionic UAUV Water-Exit Modeling

#### 2.7.1. Design Concept

In nature, certain organisms traverse the water surface with remarkable agility; flying fish are the most representative example, whose distinctive morphology confers highly efficient water-entry and water-exit performance.

Before water exit, a flying fish first reorients beneath the surface, folding its pectoral and pelvic fins tightly against the body; it then executes vigorous caudal-fin beats to generate substantial thrust that propels the body clear of the water, achieving takeoff speeds up to 18 m/s.

Upon breaching, the flying fish immediately deploys its broad, elongated pectoral fins, forming a planform akin to a fixed-wing unmanned aircraft—with a fuselage, wings, a horizontal tail, and a vertical tail. This configuration markedly increases the lifting area, enabling glides at speeds of 10–20 m/s [34].

During water entry, the flying fish folds its pectoral fins and relies on inertia to complete the maneuver, thereby reducing impact loads. It typically penetrates the surface water at a shallow incidence of 0–30°. When threatened, however, it can rapidly reorient and enter at a much steeper angle of 60–90°, facilitating rapid submergence and increasing its chances of survival.

#### 2.7.2. Bionic Modelling

**Figure 4:**
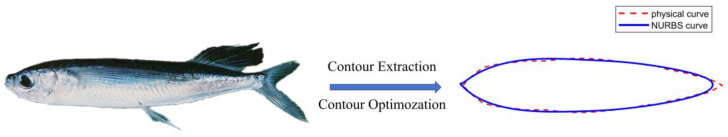
Establishment of bionic UAUV contour curves.

Based on the shape of the flying fish, the bioinspired body contour was constructed using the Non-Uniform Rational B-Splines (NURBS) method, as shown in Figure 4. The NURBS method offers excellent flexibility and accuracy in handling complex body geometries.

The NURBS curve is defined by the following formulation:

(10) $$C(u)=\frac{\sum_{i=1}^{k}N_{i,n}(u)w_{i}P_{i}}{\sum_{i=1}^{k}N_{i,n}(u)w_{i}}=\sum_{i=1}^{k}R_{i,n}(u)P_{i}$$

Here, $P_{i}$ denotes the control vertex, k denotes the number of control points, $R_{i,n}(u)$ refers to the rational basis function, and $w_{i}$ represents the weight associated with each point.

The NURBS surface is defined by the following formulation:

(11) $$S(u,v)=\sum_{i=1}^{k}\sum_{j=1}^{l}R_{i,j}(u,v)P_{i,j}$$

(12) $$R_{i,j}(u,v)=\frac{N_{i,n}(u)N_{j,m}(v)w_{i,j}}{\sum_{p=1}^{k}\sum_{q=1}^{l}N_{p,n}(u)N_{q,m}(v)w_{p,q}}$$

Here, $P_{i,j}$ represents the control vertex, $N_{p,n}(u)$ represents the n-th degree B-spline basis function in the u-direction, $w_{i,j}$ represents the weight associated with each point, and $N_{q,m}(v)$ is the m-th degree B-spline basis function in the v-direction.

#### 2.7.3. Results of Bionic Modeling and Water-Exit Process

**Figure 5:**
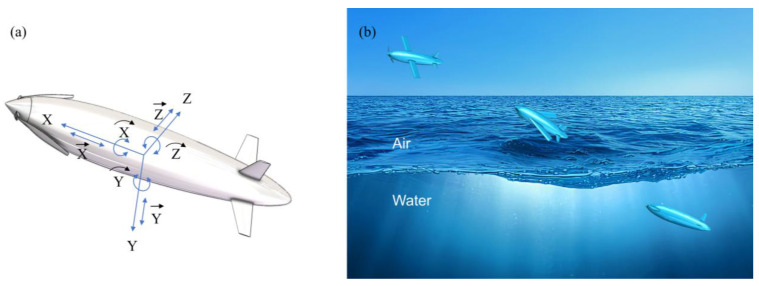
Schematic illustration of the structure and water-exit process of the bionic UAUV. (**a**) Schematic illustration of the structure of the bionic UAUV during water exit. (**b**) Schematic illustration of the bionic UAUV water-exit process.

Based on the optimized body contour, a bionic UAUV model for water exit was developed, as shown in Figure 5a. The model incorporates a front-mounted propeller, wings, and tail fins. During the water-exit process, the front-mounted propeller and wings can be folded to lie close to the body, simulating the flying fish’s water-exit behavior. The water-exit process of the bionic UAUV is illustrated in Figure 5b. Subsequently, unsteady numerical simulations were conducted to analyze the forces acting on the bionic UAUV during its water-exit process.

### 2.8. Grid Independence Verification

**Figure 6:**
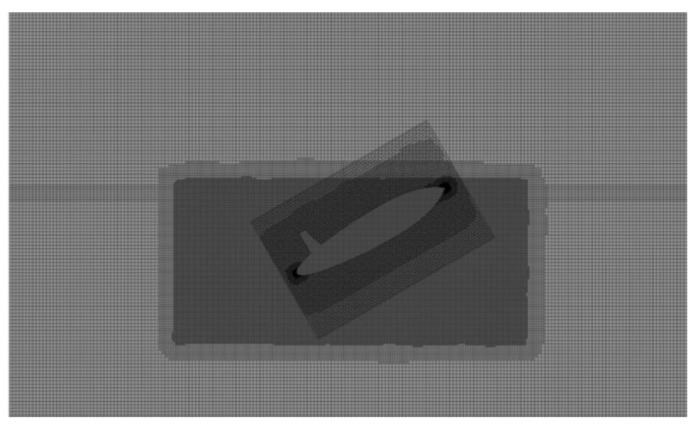
Meshing for the water-exit simulations of the bionic UAUV.

The water-exit process of the bionic UAUV was validated for an exit velocity of 8 m/s and an exit angle of 30°. The impact of mesh resolution on the results was evaluated using three different mesh densities: 4.46 million, 8.51 million, and 13.40 million cells. The mesh configurations are shown in Figure 6.

**Figure 7:**
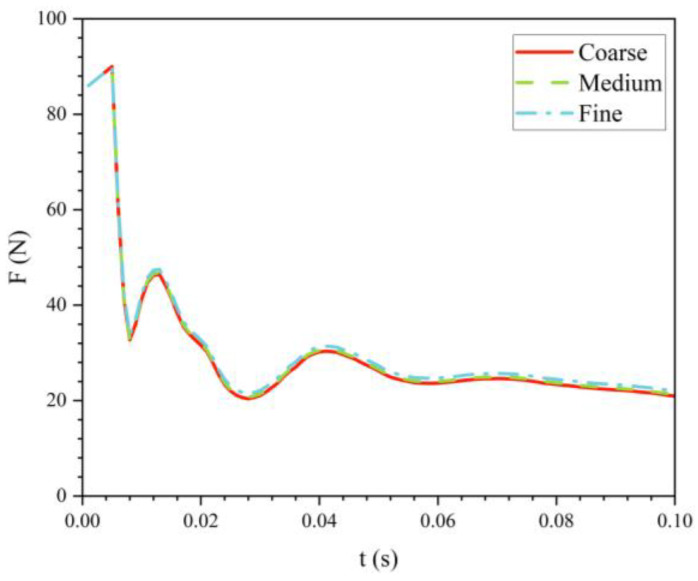
Variation of the water-exit drag over time for the bionic UAUV under different mesh densities.

Figure 7 illustrates the variation of drag over time for the bionic UAUV at a water-exit velocity of 8 m/s and an exit angle of 30° under three different mesh densities. At t = 0.013 s, the drag deviations between the coarse and medium meshes compared with the fine mesh were 1.15% and 0.41%, respectively. At t = 0.042 s, the deviations increased to 4.88% and 3.31% for the coarse and medium meshes, respectively.

**Figure 8:**
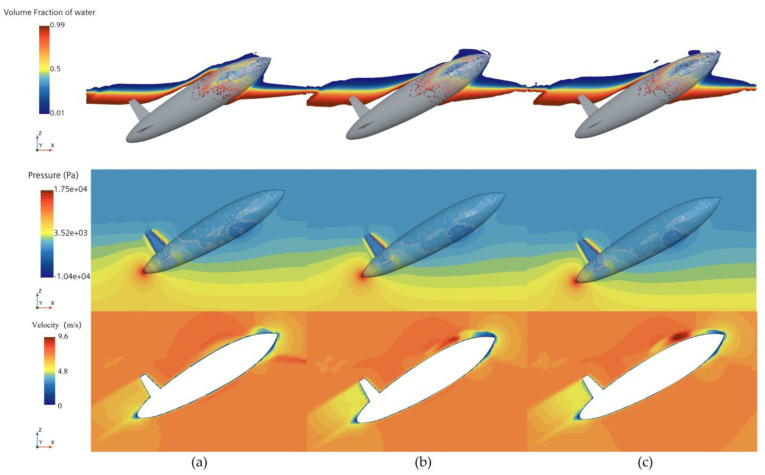
Phase and pressure distribution during the water exit of the bionic UAUV under different mesh densities. (**a**) Coarse mesh; (**b**) Medium mesh; (**c**) Fine mesh.

Figure 8 presents the phase and pressure distributions at t = 0.035 s. The phase distribution shows that the disturbances caused by the water exit of the bionic UAUV lead to significant splashing in front of the upper tail fin. Compared with the coarse mesh, the medium mesh captures the air–water interface more accurately, with the resulting phase distribution being closer to the fine mesh results. Additionally, both the medium and fine meshes demonstrate stronger robustness in capturing unsteady disturbances at the free surface. The corresponding pressure distribution results indicate only minor differences between the meshes, with all three mesh densities accurately capturing the main features of the pressure distribution. Figure 8 also presents the velocity contours to illustrate the representative flow-field features during the water-exit process.

From the numerical simulation results, it is evident that the medium mesh captures the variations in the bionic UAUV’s water-exit process more effectively than the coarse mesh, with minimal differences compared with the fine mesh results. Therefore, considering the trade-off between computational accuracy and cost, a medium-density mesh was selected for the subsequent numerical simulation analyses of the bionic UAUV’s water-exit process.

## 3. Results and Discussion

### 3.1. Effect of Foldable Propeller on Water-Exit Drag

To investigate the impact of foldable propellers on water-exit drag, simulations were conducted for the bionic UAUV in both the deployed and folded propeller states, under a water-exit angle of 30°and an exit velocity of 8 m/s.

**Figure 9:**
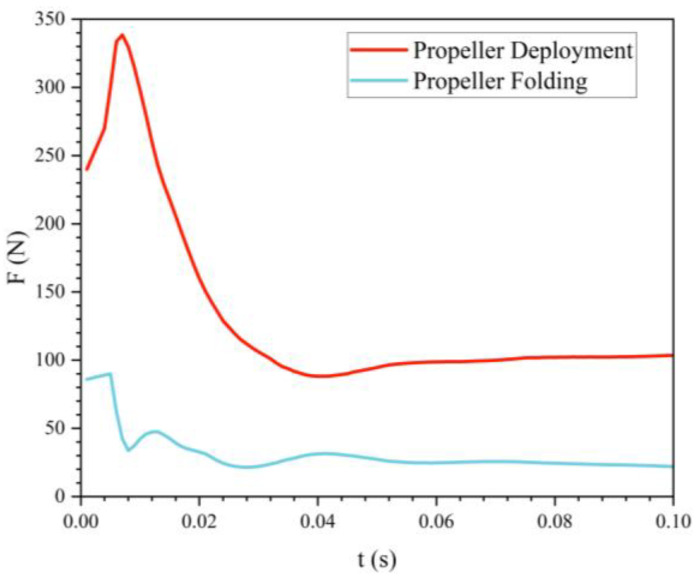
Drag of the bionic UAUV during water exit under different propeller configurations.

Figure 9 illustrates the variation of drag with time during the water-exit process of the bionic UAUV under different propeller states. The results indicate that the drag generated when the propeller is deployed is significantly higher than when the propeller is folded, with the peak drag occurring later in the deployed state. This suggests that the bionic UAUV experiences greater resistance during water exit when the propeller is in the deployed state.

**Figure 10:**
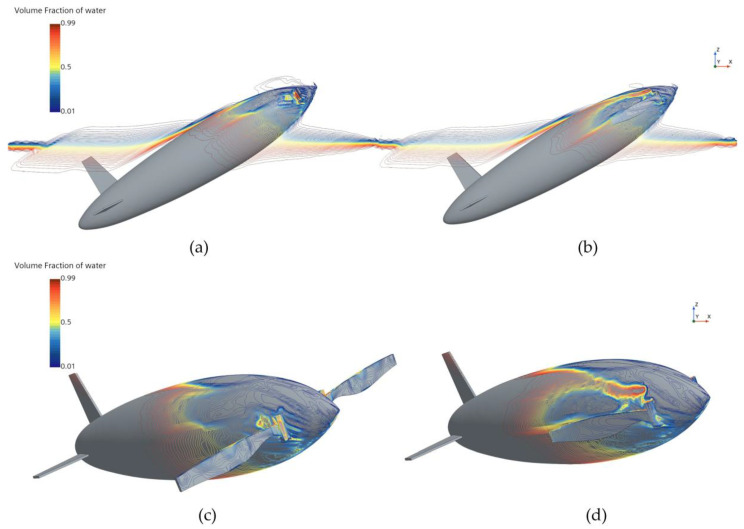
Phase distribution of the bionic UAUV water-exit process under different propeller configurations. (**a**) Propeller deployment; (**b**) Propeller Folding; (**c**) Propeller deployment; (**d**) Propeller Folding.

**Figure 11:**
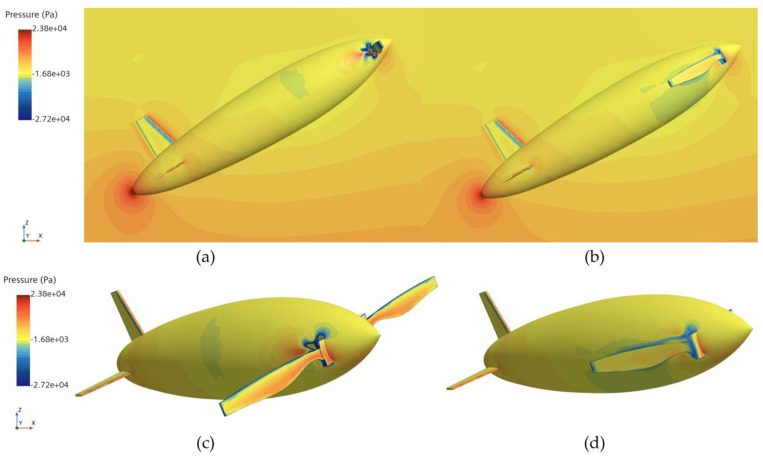
Pressure distribution during the bionic UAUV water exit under different propeller configurations. (**a**) Propeller deployment; (**b**) Propeller Folding; (**c**) Propeller deployment; (**d**) Propeller Folding.

Figure 10 and Figure 11 show the phase and pressure distributions during the water-exit process of the bionic UAUV under both propeller states. In the deployed state, the flow dynamics during water exit are more complex, as the deployed propeller obstructs the water flow, causing pressure to concentrate on the propeller blades. Excessive pressure concentration can damage the propeller blades and adversely affect the UAUV’s water-exit motion. In contrast, when the propeller is folded, the reduced interference with the flow minimizes pressure concentration, thereby alleviating potential damage to the propeller blades and lowering the risk of structural damage during the water-exit process. Therefore, the folded propeller strategy was chosen for simulating the bionic UAUV’s water-exit process in this study.

### 3.2. Effect of Water-Exit Velocity on Water-Exit Drag

To investigate the effect of exit velocity on water-exit drag, the bionic UAUV’s water-exit process was simulated at exit angles of 30°, 45°, and 60°. During the water-exit process, the bionic UAUV undergoes straight-line translation along the specified exit angle.

**Figure 12:**
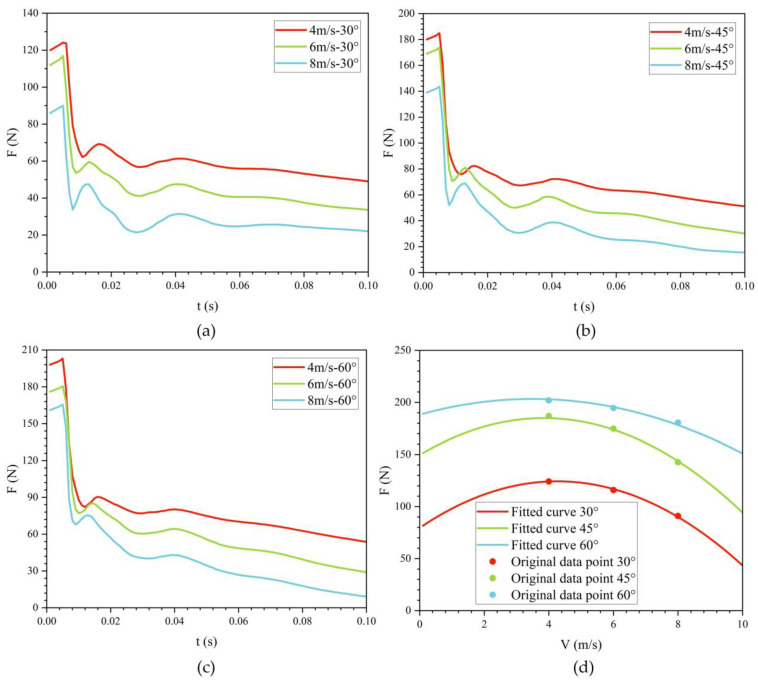
Variation of drag for the bionic UAUV at different water-exit velocities over time. (**a**) θ = 30°; (**b**) θ = 45°; (**c**) θ = 60°; (**d**) Fitted curve.

Figure 12 shows the effect of exit velocity on drag variation with time at three different exit angles. During the water-exit process, the bionic UAUV experiences three drag peaks, occurring when the front, folded propeller, and tail fin make contact with the water surface, respectively. The results indicate that as the exit velocity increases, the water-exit drag gradually decreases.

When the exit angle is 30°, the maximum water-exit drag at exit velocities of 4 m/s and 6 m/s is 1.379 and 1.299 times that at 8 m/s, respectively. At an exit angle of 45°, the maximum water-exit drag at 4 m/s and 6 m/s is 1.287 and 1.210 times that at 8 m/s, respectively. At an exit angle of 60°, the maximum water-exit drag at 4 m/s and 6 m/s is 1.137 and 1.067 times that at 8 m/s, respectively. As the exit angle increases, the ratio of maximum drag between high-speed and low-speed water exit gradually decreases. Simultaneously, the peak water-exit drag increases with the increase in exit angle.

The variation of drag with time at different exit velocities is primarily influenced by the dynamic interaction between the vehicle, water, and air during the water-exit process. When the exit velocity increases, the drag initially increases due to the higher momentum transfer between the vehicle and the surrounding fluid. However, as the exit velocity further increases, the drag starts to decrease because the vehicle exits the water more quickly, reducing the duration of the fluid interaction and the associated resistance.

This phenomenon can be explained theoretically by the relationship between the drag force and the velocity-dependent factors, such as the pressure drag and skin friction. The drag increases with higher exit velocities, particularly when the vehicle is moving through the interface between water and air. However, as the velocity increases further, the skin friction (due to turbulence and shear stress) becomes less significant, leading to a reduction in overall drag.

Additionally, the relationship between exit velocity and drag is influenced by the vehicle’s hydrodynamic characteristics, including the angle of attack and the wake formation behind the vehicle. At higher velocities, the wake becomes more stable, leading to a reduction in drag due to less turbulent shedding from the body.

Table 1 lists the three drag peaks during the water-exit process at an exit angle of 30°. In conjunction with Figure 12, it is evident that as the exit velocity increases, the occurrence times of all three peaks advance, and their magnitudes decrease progressively.

To examine the influence of exit velocity on water-exit drag, the maximum drag was determined for a range of operating conditions, and its dependence on exit velocity was analyzed. As shown in Figure 12d, the relationship is well approximated by a quadratic function: with increasing exit velocity, the peak drag first rises and then declines. Consequently, selecting either relatively low or relatively high exit velocities yields lower peak drag. In this study, an exit velocity of 8 m/s is more favorable for the bionic UAUV’s water exit than 4 m/s or 6 m/s.

**Figure 13:**
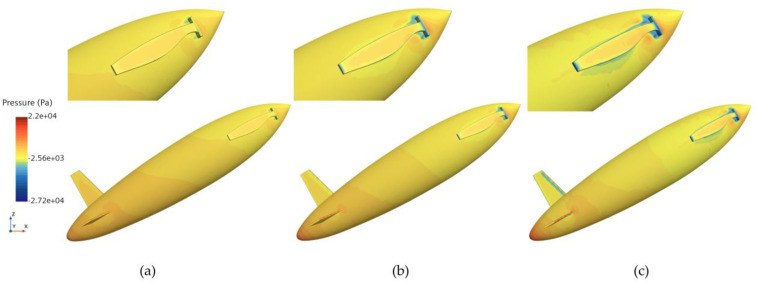
Pressure distribution at the first drag peak for the bionic UAUV at three different water-exit velocities. (**a**) v = 4 m/s; (**b**) v = 6 m/s; (**c**) v = 8 m/s.

**Figure 14:**
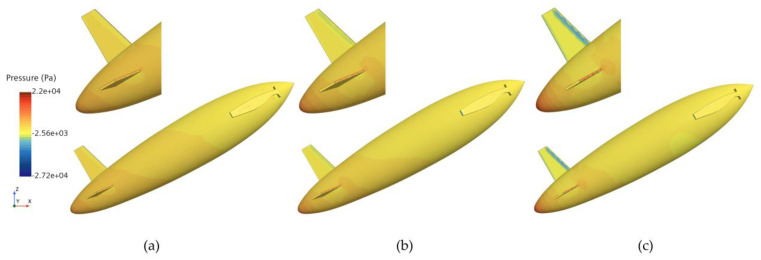
Pressure distribution at the second drag peak for the bionic UAUV at three different water-exit velocities. (**a**) v = 4 m/s; (**b**) v = 6 m/s; (**c**) v = 8 m/s.

**Figure 15:**
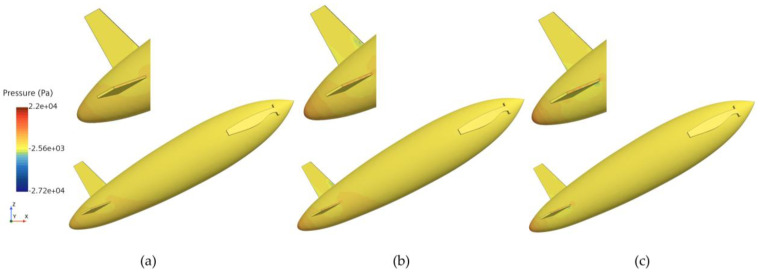
Pressure distribution at the third drag peak for the bionic UAUV at three different water-exit velocities. (**a**) v = 4 m/s; (**b**) v = 6 m/s; (**c**) v = 8 m/s.

Figure 13, Figure 14 and Figure 15, respectively, present the pressure distributions at the first, second, and third drag peaks for an exit angle of 30°, evaluated at three different exit velocities.

### 3.3. Effect of Water-Exit Angle on Water-Exit Drag

**Figure 16:**
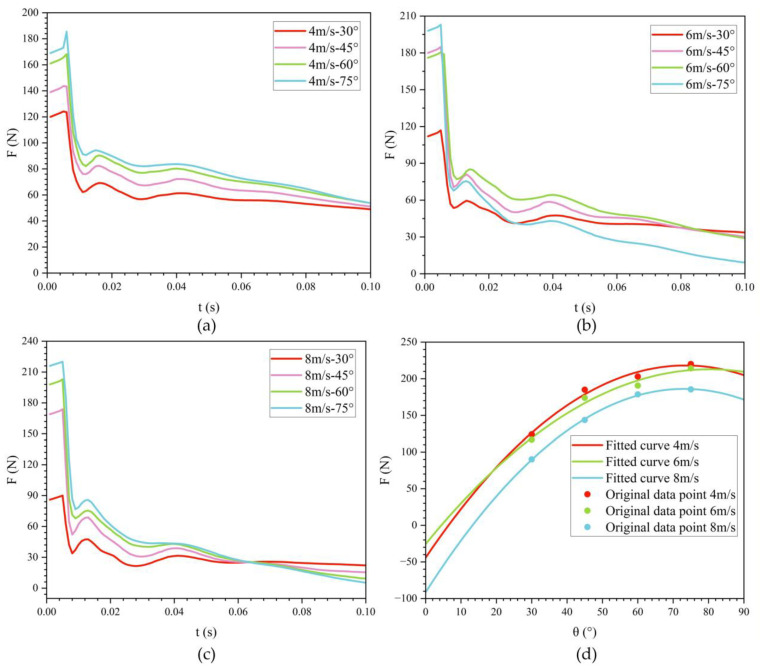
Variation of drag for the bionic UAUV at different water-exit angles over time. (**a**) v = 4 m/s; (**b**) v = 6 m/s; (**c**) v = 8 m/s; (**d**) Fitted curve.

To assess the influence of exit angle on water-exit drag, the bionic UAUV’s water-exit process was simulated at three exit velocities: 4 m/s, 6 m/s, and 8 m/s. To better investigate angle effects, four exit angles were considered: 30°, 45°, 60°, and 75°. Figure 16 presents the time histories of drag for each exit velocity across these angles, and Table 2 lists the corresponding maximum drag values. Figure 16d shows a fitted curve describing the relationship between exit angle and maximum drag. The analysis indicates that the maximum drag increases with exit angle, reaches a maximum near 80°, and then decreases, exhibiting an approximately quadratic trend.

In Figure 16b, the drag history for the 30° exit angle at 6 m/s shows smaller fluctuations compared with the curves for the other exit angles (45°, 60°, and 75°). This can be attributed to the smoother and more gradual transition of the vehicle from water to air at smaller exit angles. At 30°, the vehicle remains in contact with the water for a longer period, and the flow separation is less abrupt compared with higher exit angles. As a result, the drag increases more gradually and experiences smaller fluctuations throughout the water-exit process. This is because, at smaller exit angles, the flow around the vehicle tends to be more stable, leading to a more continuous change in drag. In contrast, at higher exit angles (45°, 60°, and 75°), the vehicle exits the water more rapidly, causing faster flow separation and the development of stronger wake turbulence. This leads to larger fluctuations in drag as the vehicle experiences more dynamic interactions with the fluid during its exit process.

**Figure 17:**
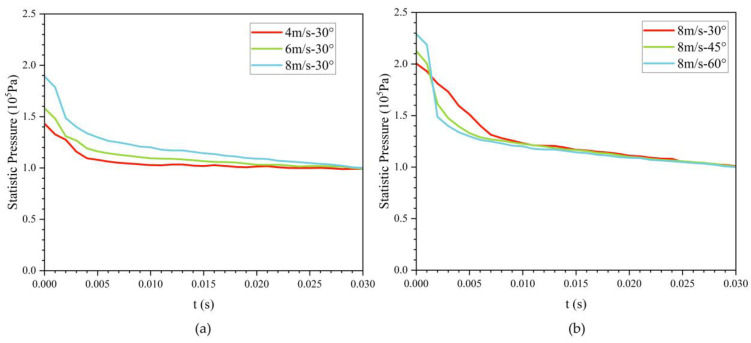
Temporal evolution of the bionic UAUV’s maximum surface pressure during the water-exit process. (**a**) 30°; (**b**) 8 m/s.

Simulation results across exit angles show that the bionic UAUV experiences the largest water-exit drag at an exit angle of 75°. Specifically, at an exit velocity of 4 m/s, the drag reaches 220.041 N at t = 0.007 s. Therefore, to safeguard structural integrity, large exit angles should be avoided. Figure 17 depicts the time evolution of the maximum surface pressure during water exit. Owing to the bioinspired streamlined fuselage, the surface pressure drops rapidly during the water-exit process and then gradually levels off.

**Figure 18:**
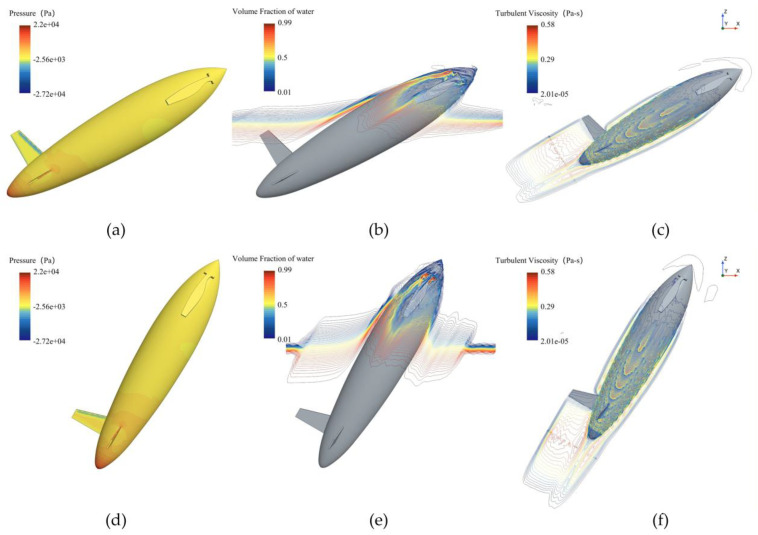
Pressure distribution, phase distribution, and turbulent viscosity of the bioinspired UAUV at the second water-exit drag peak (8 m/s). (**a**) Pressure distribution (θ = 30°); (**b**) Phase distribution (θ = 30°); (**c**) Turbulent viscosity (θ = 30°); (**d**) Pressure distribution (θ = 60°); (**e**) Phase distribution (θ = 60°); (**f**) Turbulent viscosity (θ = 60°).

Figure 18 shows the surface turbulent viscosity, phase, and pressure distributions for the bionic UAUV at the second drag peak (v = 8 m/s). At the smaller exit angle (θ = 30°), the surface pressure is more uniform, the phase field in the wake is relatively stable, turbulence levels are low, and the water exit proceeds smoothly. At the larger exit angle (θ = 60°), high-pressure regions intensify, the phase field in the wake becomes disordered, and both turbulent viscosity and dissipation increase, leading to markedly higher water-exit drag and stronger flow unsteadiness.

**Figure 19:**
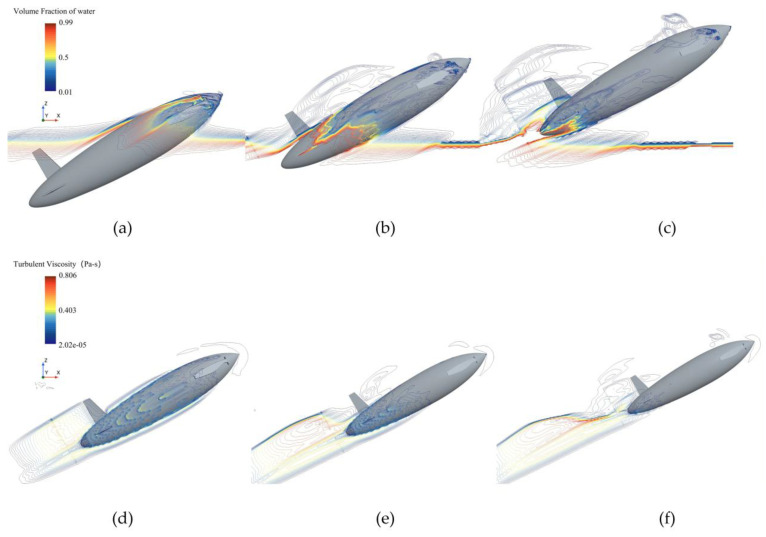
Variation of phase distribution (**a**–**c**) and turbulent viscosity (**d**–**f**) with time during the water exit of the bionic UAUV (8 m/s, 30°). (**a**) Contact with the water surface; (**b**) Incompletely exiting the water; (**c**) Full exit; (**d**) Contact with the water surface; (**e**) Incompletely exiting the water; (**f**) Full exit.

As revealed by the phase and turbulent viscosity distribution in Figure 19, the streamlined, bioinspired configuration results in a smooth, displacement-type disturbance of the free surface. Owing to the relatively low exit velocity and the streamlined body, this disturbance does not trigger cavitation; however, it markedly influences the surface pressure distribution, flow separation, and transition over the bionic UAUV.

### 3.4. Effect of Drag on Pitch Characteristics

#### 3.4.1. Single-Degree-of-Freedom Pitch Analysis During Water Exit

**Figure 20:**
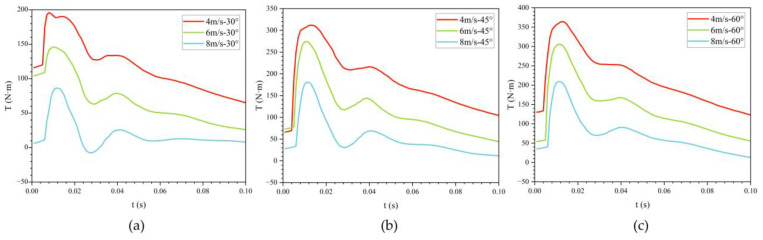
Variation of pitch moment during water exit of the bionic UAUV over time. (**a**) 30°; (**b**) 45°; (**c**) 60°.

Figure 20 presents the time histories of pitching moment during the water exit of the bionic UAUV at three exit angles—30°, 45°, and 60°—and three exit velocities—4 m/s, 6 m/s, and 8 m/s. Based on the numerical results, the influence of water-exit drag on the vehicle’s pitch characteristics is examined.

Figure 20 provides a direct visualization of the pitching moment time histories at different exit velocities. As depicted in Figure 20c, the pitching moment of the bionic UAUV at a 60° water angle exhibits two distinct peaks. The first peak arises almost instantaneously, followed by a decrease. The second peak coincides with the emergence of the tail fin, after which the moment gradually decays. At lower exit velocities, the fluctuation amplitude is larger. After complete emergence, the pitching moment tends toward zero.

Figure 20a,b show the time histories of pitching moment during water exit at 30° and 45°. As the exit angle decreases, the fluctuation amplitude diminishes, and the peak values are markedly reduced. Nevertheless, the overall evolution remains similar to that observed for the 60° case.

In conjunction with the pressure and phase analyses of the bionic UAUV’s water-exit process in Figure 18, larger exit angles produce richer surface-flow phenomena and more pronounced liquid splashing. These effects significantly influence the water-exit drag and the pitching moment characteristics. To maintain a stable attitude and reduce water-exit drag, the optimal exit angle for the bionic UAUV examined here is 30°.

#### 3.4.2. Multi-Degree-of-Freedom Pitch Analysis During Water Exit

During the oblique water exit of the bionic UAUV, moments induced by asymmetric forces exert a pronounced influence; these unsteady moments can substantially alter the vehicle’s exit attitude. Therefore, to more realistically simulate and analyze the angular evolution during water exit, pitch motion about the transverse axis through the UAUV’s center of gravity (CG) was superimposed on the translational motion.

A multi-degree-of-freedom numerical simulation of the bionic UAUV’s water exit at an exit angle of 30° and an exit velocity of 8 m/s was performed. An active control strategy was implemented to reduce the peak pitching moment, thereby mitigating its influence on the bionic UAUV’s angular evolution during exit.

**Figure 21:**
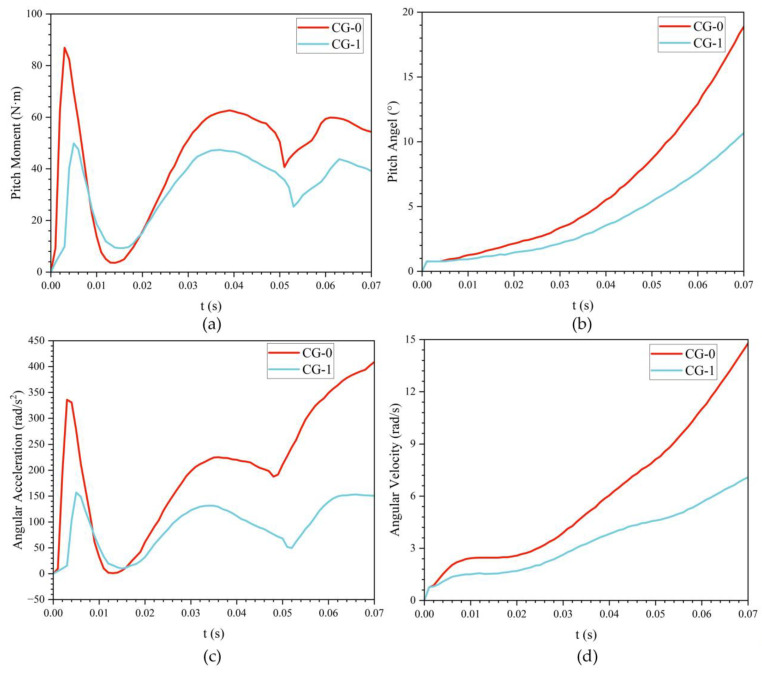
Variation of pitching characteristics during water exit of the bionic UAUV at different centers of gravity over time. (**a**) Pitch moment versus time; (**b**) Pitch angle versus time; (**c**) Angular acceleration versus time; (**d**) Angular velocity versus time.

**Figure 22:**
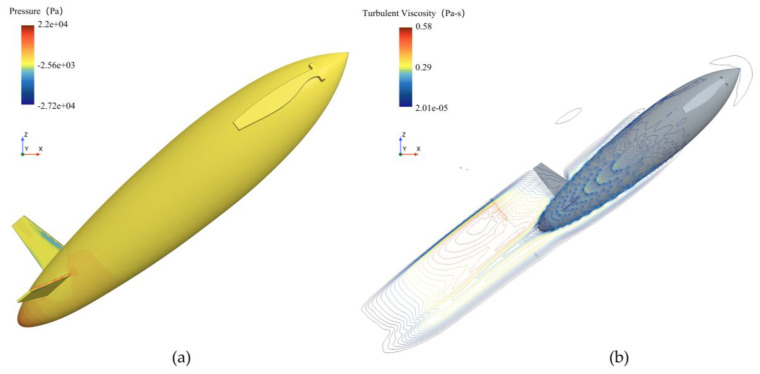
Multi-degree-of-freedom water-exit simulation of the bionic UAUV. (**a**) Surface Pressure; (**b**) Surface Turbulent Viscosity.

Figure 21 shows the time histories of angular acceleration, pitching moment, angular velocity, and pitch angle for the bionic UAUV with different center of gravity locations. Figure 22 provides a schematic of the multi-degree-of-freedom water-exit simulation. Across center of gravity positions, these quantities exhibit similar evolutionary trends. Smaller pitch angle excursions promote greater exit stability, higher emergence height, and improved attitude control. To mitigate the nose-up moment induced by water-exit drag, either active or passive control strategies may be employed. The single-degree-of-freedom water exit can be regarded as an idealized case that neglects pitch motion; by enforcing real-time control to maintain the pitch angle at 0° throughout the exit, this ideal condition can be approximated.

### 3.5. Drag Model for Different Water-Exit Conditions

**Figure 23:**
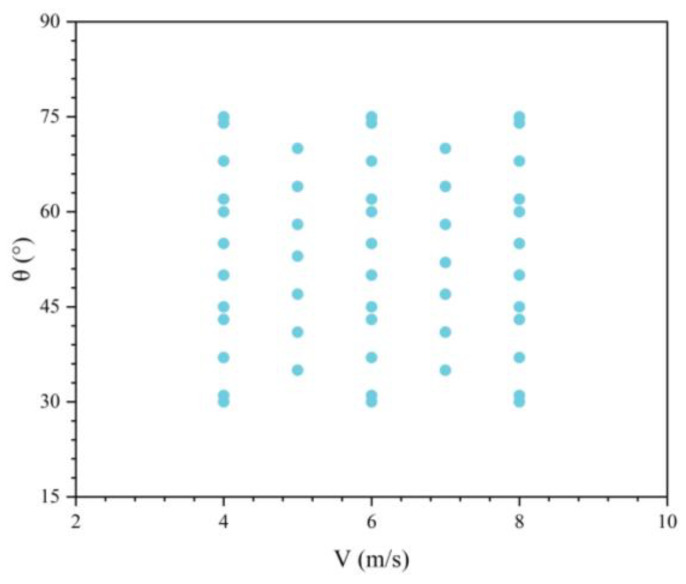
Distribution of the 50-case dataset used for the predictive models.

Predictive models were constructed to characterize the drag variation of the UAUV across different water-exit states, using a dataset of 50 numerical cases from the water-exit simulations presented in Figure 23. Specifically, water-exit drag prediction models based on response surface methodology (RSM) and support vector regression (SVR) were developed, and their accuracy was compared. The results provide a methodological reference for future studies on drag prediction and design optimization.

The predictive models were evaluated using the coefficient of determination (R^2^), mean squared error (MSE), root mean square error (RMSE), and mean absolute error (MAE). A model with a larger R^2^ (closer to 1) and smaller MSE, RMSE, and MAE values indicates a better fit and higher predictive accuracy. The definitions of these metrics are as follows:

(13) $$R^{2}=1-\frac{\sum_{i=1}^{n}{\left(y_{i}-{\hat{y}}_{i}\right)}^{2}}{\sum_{i=1}^{n}{\left(y_{i}-\bar{y}\right)}^{2}}$$

(14) $$R^{2}=1-\frac{\sum_{i=1}^{n}{\left(y_{i}-{\hat{y}}_{i}\right)}^{2}}{\sum_{i=1}^{n}{\left(y_{i}-\bar{y}\right)}^{2}}$$

(15) $$RMSE=\sqrt{\frac{1}{n}{\sum_{i=1}^{n}\left(y_{i}-{\hat{y}}_{i}\right)}^{2}}$$

(16) $$MAE=\frac{1}{n}\sum_{i=1}^{n}\left|y_{i}-{\hat{y}}_{i}\right|$$

Here, n is the total number of samples, $y_{i}$ is the actual value, ${\hat{y}}_{i}$ is the predicted value, and $\bar{y}$ is the mean of the actual values.

Equation (17) presents the relationship between the maximum water-exit drag F, water-exit angle θ and water-exit velocity v, as derived from the RSM–based predictive model.

(17) $$\begin{array}{l} F=-159.5404+12.6457v+13.6442\theta -2.0873v^{2}-0.1876\theta^{2}-0.0043v\theta \\ -0.013v^{3}+0.001\theta^{3}+0.0108v^{2}\theta -0.0008v\theta^{2} \end{array}$$

**Figure 24:**
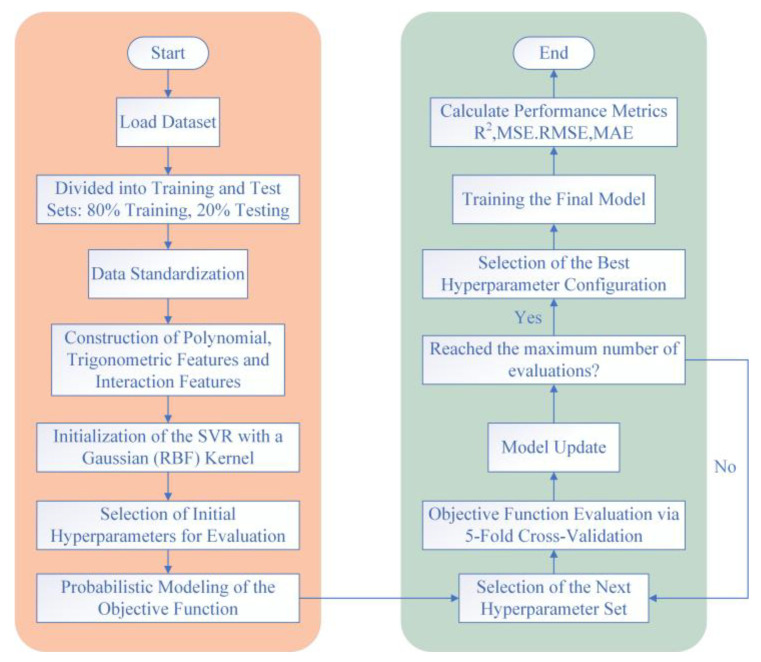
Workflow of the support vector regression (SVR) model.

Figure 24 outlines the workflow of the SVR model used in this study. The dataset is first split 8:2 into training and test sets, followed by data standardization. On this basis, polynomial and interaction features are constructed from the baseline variables, and trigonometric features of the attitude angle are added to capture angular periodicity. Bayesian (probability-based) global optimization is then employed to iteratively search and evaluate the hyperparameters—the penalty parameter C, the kernel scale γ, and the ε parameter of the ε-insensitive loss. This proceeds in a loop: at each iteration, a surrogate probabilistic model proposes the next hyperparameter candidate; performance is assessed via 5-fold cross-validation and used to update the surrogate. The loop continues until a preset maximum number of evaluations is reached, after which the best-performing configuration is selected to train the final model. Finally, the model is quantitatively evaluated using R^2^, MSE, RMSE, and MAE.

**Figure 25:**
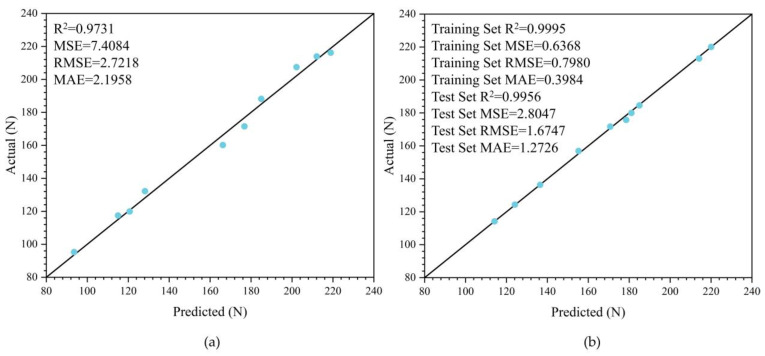
Accuracy assessment of the water-exit drag prediction models based on RSM and SVR. (**a**) RSM-based water-exit drag model; (**b**) SVR-based water-exit drag model.

Figure 25 presents the accuracy assessment of the water-exit drag models built with the RSM and SVR. The RSM-based model provides generally good predictions across different exit states, but the SVR-based model attains higher accuracy: R^2^ improves from 0.9731 to 0.9956 (relative gain ≈ 2.31%), RMSE decreases from 2.7218 to 1.6747 (relative reduction ≈ 38.5%), and MAE drops from 2.1958 to 1.2726 (relative reduction ≈ 42.0%). These results indicate that, for water-exit processes with pronounced nonlinearity and coupled-variable effects, SVR with a Gaussian (RBF) kernel and Bayesian hyperparameter optimization captures the complex input–output mapping more effectively.

In summary, the RSM-based water-exit drag model provides explicit, physically interpretable empirical formulas suitable for rapid estimation and sensitivity analysis, whereas the optimized SVR-based water-exit drag model has higher predictive accuracy across different water-exit states and is better suited as the primary model for engineering computations and control strategies. Used together, the two offer a favorable balance between accuracy and interpretability.

The water-exit drag prediction model developed herein is applicable to the analysis of water-exit processes for a wide range of UAUVs. Once the actual water-exit state of a UAUV is specified, the model can be used to estimate the loads it experiences. During fabrication of physical prototypes, these estimated loads provide references for structural strength design. In practice, the allowable structural load should exceed the load estimated by the water-exit drag model and be multiplied by an appropriate safety factor.

## 4. Conclusions

1. An integrated numerical framework and solution procedure—combining a modified SST *k-ω* turbulence model, a volume of fluid interface-capturing scheme, overset grids, and six degrees of freedom rigid body dynamics—was established. Validation was conducted across multiple operating conditions in the two dimensions of water-exit velocity and water-exit angle. The results indicate strong robustness and high fidelity in reproducing the bionic UAUV’s water-exit process and its key features.
2. The bionic modeling of the UAUV with a front-mounted propeller is inspired by the fin-folding behavior of flying fish during water exit. The overall configuration adopts a streamlined layout; with the propeller folded, the vehicle exhibits superior water-exit performance. Under conditions of water-exit velocity V ≥ 8 m/s and water-exit angle θ ≤ 30°, the maximum water-exit drag is less than 90.004 N.
3. The influences of the front-mounted propeller configuration, water-exit velocity, and water-exit angle on water-exit drag were quantitatively evaluated. The results indicate that folding the front-mounted propeller significantly reduces exit drag and mitigates high-pressure concentration on the blades. The peak water-exit drag shows a pronounced quadratic dependence on both water-exit velocity and water-exit angle. To ensure safe emergence, water exit with the front-mounted propeller deployed should be avoided, as should very low exit velocities (e.g., 4 m/s) and excessively large exit angles (e.g., 75°).
4. The turbulence characteristics during the bionic UAUV’s water exit are correlated with the peak oscillations of the exit drag. The streamlined, bioinspired configuration reduces the influence of the pressure distribution on pitch response, resulting in only minor pitch-angle excursions during exit.
5. Predictive models of water-exit drag for the bionic UAUV, constructed using response surface methodology (RSM) and support vector regression (SVR), effectively elucidate the relationship between water-exit state and water-exit drag. This approach provides practical bioinspired design guidance and feasible analysis strategies for UAUV development.
6. The key contributions of this work are twofold: first, this study provides a detailed analysis of the controllable water-exit behavior of the bioinspired UAUV, which informs effective design strategies for optimizing water-exit performance; second, comprehensive predictive models for water-exit drag are developed, offering valuable insights for the design and analysis of bioinspired UAUVs. Future research will focus on examining the behavior of uncontrolled water-exit motion under more complex conditions, such as wave environments, to further improve the performance and predictability of UAUVs.

## Figures and Tables

**Table 1:** Relationship between peak drag and water-exit velocity at a 30° water-exit angle.

The Water-Exit Velocity (m/s)	4	6	8
Time of the first peak value (s)	0.007	0.006	0.005
The first peak value(N)	124.139	116.910	90.000
Time of the second peak value (s)	0.016	0.014	0.013
The second peak value (N)	69.194	59.539	47.504
Time of the third peak value (s)	0.045	0.043	0.041
The third peak value (N)	61.320	47.520	31.449

**Table 2:** Maximum drag across various water-exit angles at specified velocities.

Water-Exit Velocity	Water-Exit Angle			
	30°	45°	60°	75°
4 m/s	124.139 N	184.884 N	202.992 N	220.041 N
6 m/s	116.910 N	173.825 N	190.546 N	214.170 N
8 m/s	90.004 N	143.611 N	178.546 N	185.440 N

## Data Availability

The original contributions presented in this study are included in the article. Further inquiries can be directed to the corresponding author.

## Nomenclature

**Symbol**	**Description**	**Unit**
F	Water-exit drag	N
k	Turbulent kinetic energy	m^2^/s^2^
m	Mass of UAUV	kg·m^2^
M	Inertia tensor	N·m
P	Pressure	Pa
$P_{k}$	Production term of k	model-consistent
$P_{\omega }$	Production term of ω	model-consistent
t	Time	s
$u_{i}$,$u_{j}$	Velocity components	m/s
V	Translational velocity	m/s
v	Water-exit velocity	m/s
$x_{i}$,$x_{j}$	Cartesian coordinates	m
α	Volume fraction	—
β,$\beta^{\prime }$	SST model constants	—
μ	Laminar viscosity	Pa·s
$\mu_{T}$	Turbulent viscosity	Pa·s
$\mu_{m}$	Mixture laminar viscosity	Pa·s
ρ	Density	kg/m^3^
$\sigma_{k}$	SST constant for k equation	—
$\sigma_{\omega }$, $\sigma_{\omega 2}$	SST constants for ω equation	—
ω	Specific dissipation rate	1/s
Ω	Vorticity magnitude	1/s
θ	Water-exit angle	°
